# Comparative Transcriptome Analysis of MeJA Responsive Enzymes Involved in Phillyrin Biosynthesis of *Forsythia suspensa*

**DOI:** 10.3390/metabo12111143

**Published:** 2022-11-20

**Authors:** Xiaoran Liu, Jiaqi Zhang, Hao Liu, Huixiang Shang, Xingli Zhao, Huawei Xu, Hongxiao Zhang, Dianyun Hou

**Affiliations:** 1 Agricultural of College, Henan University of Science and Technology, Luoyang 471023, China; 2 The Luoyang Engineering Research Center of Breeding and Utilization of Dao-di Herbs, Luoyang 471023, China; 3 Sanmenxia Academy of Agricultural Sciences, Sanmenxia 472000, China

**Keywords:** *Forsythia suspensa*, comparative transcriptome, MeJA treatment, phillyrin, phillyrin biosynthesis

## Abstract

*Forsythia suspensa* (Thunb.) has been widely used in traditional medicines in Asia. According to the 2020 edition of Chinese Pharmacopoeia, phillyrin is the main active ingredient in *F. suspensa*, which is effective in clearing heat, reducing swelling, and dispersing nodules. *F. suspensa* leaf is a non-toxic substance and it can be used to make a health tea. Here, we combine elicitors and transcriptomics to investigate the inducible biosynthesis of the phillyrin from the *F. suspensa*. After the fruits and leaves of *F. suspensa* were treated with different concentrations of methyl jasmonate (MeJA), the content of phillyrin in the fruits reached a peak at 200 µM MeJA for 12 h, but which was decreased in leaves. To analyze the differences in key enzyme genes involved in the phillyrin biosynthesis, we sequenced the transcriptome of *F. suspensa* leaves and fruits treated with 200 µM MeJA for 12 h. We hypothesized that nine genes related to coniferin synthesis including: *F. suspensa UDP-glycosyltransferase* (*FsUGT*); *F. suspensa 4-coumarate coenzyme CoA ligase* (*Fs4CL)*; and *F. suspensa Caffeoyl-CoA O-methyltransferase (FsCCoAOMT)* etc. The qRT-PCR analysis of genes related to phillyrin biosynthesis was consistent with RNA-seq analysis. We also investigated the dynamic changes of genes in *F. suspensa* leaves and fruits at different time points after 200 µM MeJA treatment, which laid the foundation for further study of the molecular mechanisms regulating the biosynthesis of phillyrin.

## 1. Introduction

*Forsythia suspensa* (Thunb.) is utilized as a common traditional medicine in China, Japan, Korea, and many European countries. It is called ‘Lianqiao’ in China [1]. Based on the different harvest time, *F. suspensa* fruits can be classified into ‘Qingqiao’ and ‘Laoqiao’ forms. In folk medicine, the extract of the dried fruit has long been used to treat a variety of diseases, such as inflammation, pyrexia, gonorrhea, tonsillitis, and ulcers [2]. *F. suspensa* fruit is also the main active ingredient in many widely used classic Chinese patent medicine prescriptions, such as Shuanghuanglian injections [3] and Lianhua Qingwen granules [4]. Lianhua Qingwen is also recommended for the treatment of COVID-19 [5]. The dried ripe fruit of *F. suspensa* has also been prescribed for the treatment of diabetes in China [6]. In addition to the medicinal value of *F. suspensa* fruit, it has also been reported in recent years that the extract of *F. suspensa* leaves has antibacterial and other pharmacological effects [7]. In folk medicine, *F. suspensa* leaves are also used as tea [8]. Therefore, it is of great significance to study the secondary metabolites and their synthetic pathways in *F. suspensa*. In recent years, many active ingredients have been identified in *F. suspensa*, such as phenylethanoid glycosides, lignans, flavonoids, terpenes, and volatile oils [9]. Lignan and phenylethanol glycoside are the two main representative characteristic components in *F. suspensa*; more than 30 lignans and lignan glycosides, including (+)-forsythin, have been isolated from *F. suspensa* alone [10]. Phillyrin (also named forsythin), a kind of lignan substance, mitigates apoptosis and oxidative stress [11], has anti-viral and anti-inflammatory activity [12], and improves insulin resistance [13]. The biosynthesis of lignan (including phillyrin) consists of three stages: coniferyl alcohol, the precursor of lignan, is synthesized through the phenylpropanoid pathway; then, structurally diverse lignans are synthesized from coniferyl alcohol; and finally, lignans are modified by glycosylation to form lignan glycosides, which are accumulated and stored in plant cells and tissues [14].

The phenylpropane biosynthesis pathway shares some intermediates with the lignan biosynthesis pathways [15]. Based on previous study, phenylalanine ammonia-lyase enzyme (PAL), coumarate 3-hydroxylase (C3H), 4-coumarate CoA ligase (4CL), caffeoyl o-methyltransferase (COMT), caffeoyl-CoA o-methyltransferase (CCoAOMT), cinnamoyl-CoA reductase (CCR), cinnamyl alcohol dehydrogenase (CAD), dirigent protein (DIR), o-methyltransferase (OMT), and udp-glycosyltransferase (UGT), are key enzymes of the phillyrin-related secondary metabolism in plants. PAL is the enzyme that catalyzes the first step reaction of phenylpropanoid metabolism, connects the biological primary metabolism, and is the key and rate-limiting enzyme of phenylpropanoid metabolism [16]. C3H catalyzes the formation of caffeic acid from coumaric acid, which is methylated to form ferulic acid [17]. The enzyme 4CL activates cinnamic acid and its hydroxylated derivatives by forming the corresponding CoA thioesters, and it is one of the key enzymes in lignan biosynthesis [18]. Both COMT and CCoAOMT are substrate methylation enzymes, and lignan monomer synthesis requires a two-step methylation reaction at the 3′ and 5′ positions of the two-step methylation reaction; COMT and CCoAOMT are two methylation enzymes at different substrate levels that are associated with the specific synthesis of lignan monomers [19,20]. As an enzyme that catalyzes the first reaction of the lignan synthesis pathway, CCR is considered to be a potential control point for regulating the flow of carbon to lignans [21]. CAD catalyzes the formation of coniferyl dehyde to coniferyl alcohol, which is one of the key enzymes in lignan synthesis [14]. Although a coniferyl alcohol has yet to be identified, a dirigent protein (DIR) was shown to participate in the stereo-specific dimerization of E-coniferyl alcohol [22]. In the biological pathway for the formation of phillyrin from coniferyl alcohol, after the formation of (+)-epipinoresinol, phillygenin can be produced by oxygen methylation, possibly mediated by a specifc O-methylase [23]. Most lignans exist in plants in the form of glycosylation, which is catalyzed by UGT to generate lignan glycosides [24]. Methyl jasmonate (MeJA), a well-known exogenous inducing factor, participates in many plant processes, ranging from plant defense to growth and development [25]. As an abiotic inducer, MeJA can rapidly and selectively induce the expression of specific gene associated with specific biological process during plant secondary metabolism, thereby regulating the synthesis of secondary metabolites in plant cells [26,27]. MeJA-induced transcriptome changes have been analyzed in many different plant species including wheat and sweet basil [28,29]. In addition, genomic and transcriptomic information of *F. suspensa* is scarce in public databases. To identify the genes involved in the biosynthesis of phillyrin, the fruits and leaves of *F. suspensa* plants after 12 h of MeJA treatment were explored by transcriptional sequencing, based on the Illumina Novaseq 6000 sequencing platform, and the relevant genes in the phillyrin synthesis pathway were analyzed using Quantitative Real-Time PCR (qRT-PCR). This study provides a theoretical basis for further exploring the molecular regulation mechanism of secondary metabolites in *F. suspensa* and reveals the mechanism of the formation of phillyrin in *F. suspensa*.

## 2. Materials and Methods

### 2.1. Plant Materials and Treatment

*F. suspensa* used in this study was grown in 2021 at the *F. suspensa* medicine source base in Yiyang, Luoyang, China. In this study, fruit, and leaf tissues of *F. suspensa* plants, were treated with 50, 200, and 400 µM of MeJA. Preparation of MeJA (Sigma-Aldrich, 95%, Sigma, St. Louis, MO, USA) stock solution: 218 µL MeJA stock solution was dissolved in 2 mL absolute ethanol, then diluted with ddH_2_O to make the 10 mM MeJA solution. The high (400 µM), medium (200 µM), and low (50 µM) concentrations of MeJA treatment solution were obtained by diluting the 10 mM MeJA solution, respectively. During the experiment, final concentrations of MeJA solutions (50, 200, and 400 µM) were sprayed on the fruit and leaf surfaces of *F. suspensa* plants until the solution covered the leaf and fruit surfaces just dripping down. Control plants were sprayed with an equal volume of ethanol in ddH_2_O. For each treatment, each replicate was considered and three plants were collected. Leaves and fruits from treatments 0 h (as control), 7 h, 12 h, 24 h, 48 h were collected, frozen in liquid nitrogen, and stored at −80 °C. The *F. suspensa* fruit treated with 50 µM MeJA, 200 µM MeJA, and 400 µM MeJA were represented as M1F, M2F and M3F, respectively. The *F. suspensa* leaf treated with 50 µM MeJA, 200 µM MeJA, and 400 µM MeJA, were represented as M1L, M2L, and M3L, respectively.

### 2.2. Determination of Phillyrin Content in F. suspensa Fruits and Leaves

High performance liquid chromatography (HPLC, Agilent 1260 Infinity, Agilent Technologies Co. Ltd., Palo Alto, CA, USA) was used to determine the content of phillyrin in *F. suspensa* fruit and leaf. The MeJA-treated *F. suspensa* fruits and leaves were lyophilized and homogenized in a sterilized mortar. The extract was sonicated with 15 mL of methanol (70% *v*/*v*) for 30 min for a total of 0.5 g. The extract was cooled to room temperature and centrifuged at 4000 rpm for 10 min. The filtrate was then filtered through a 0.22 µm organic microporous membrane and stored under seal.

HPLC conditions: the mobile phase consisted of acetonitrile (A)-water (B) with gradient elution (0–40 min, 25% A) was at flow rate 1.0 mL min^−1^, column temperature 30 °C, the column was manufactured by Thermo Fisher Scientific (Waltham, MA, USA) Co., Ltd., with a diameter of 4.6 mm, a length of 250 mm, and a particle size of 5 μm. Detection wavelength 277 nm. The injection volume of the HPLC method was 10 µL. For the quantification of phillyrin, standards were used. The results were described in grams per kilogram of fresh weight. All experiments were repeated in triplicate.

### 2.3. RNA Extraction, cDNA Library Construction and Illumina Sequencing

We analyzed the content of phillyrin at different time periods after different concentrations of MeJA treatment by HPLC, and selected *F. suspensa* fruits (M2F)/leaves (M2L) treated with 200 µM MeJA for 12 h and *F. suspensa* fruits (CKF)/leaves (CKL) treated with control solution for 0 h for transcriptome sequencing including 12 samples with three replicates for each material. Total RNA was extracted from the tissue using TRIzol^®^ Reagent (Invitrogen, Carlsbad, CA, USA) (Plant RNA Purification Reagent for plant tissue) according the manufacturer’s instructions (Invitrogen) and genomic DNA was removed using DNase I (TaKara, Japan). RNA degradation and contamination was monitored on 1% agarose gels. Then, the integrity and purity of the total RNA quality was determined by 2100 Bioanalyser (Agilent Technologies Co. Ltd., Palo Alto, CA, USA) and quantified using the ND-2000 (NanoDrop Technologies, Wilmington, DE, USA). Only high-quality RNA sample (OD260/280 = 1.8~2.2, OD260/230 = 1.8~2.2, RIN = 8.8~10.0, 28S:18S = 1.4~1.8) was used to construct sequencing library. RNA purification, reverse transcription, library construction and sequencing were performed at Shanghai Majorbio Bio-pharm Biotechnology Co., Ltd. (Shanghai, China) according to the manufacturer’s instructions (Illumina, San Diego, CA, USA). Paired-end RNA-seq sequencing library was sequenced with the Illumina NovaSeq 6000 sequencer (Illumina, San Diego, CA, USA) (2 × 150 bp read length). Each sample contained three biological replicates.

### 2.4. Transcriptome Assembly and Annotation

The high-quality clean reads were obtained after removing reads containing adapters and low-quality reads. Transcriptome de novo assembly was performed using the Trinity program [30]. Functional annotation of the unigenes was carried out using BLASTx program with an E-value threshold of 1 × 10^−5^ against the NCBI non-redundant protein (Nr) datbase [31], Swiss-Prot protein database, COG database [32], and Kyoto Encyclopedia of Genes and Genomes (KEGG) database [33]. GO annotation was analyzed by Blast2GO software (Version 2.2.31+) [34]. Transcription factors (TFs) were identified using PlantTFDB database with default parameters [35].

### 2.5. Differentially Expressed Genes (DEGs) Analysis

The high-quality RNA reads were counted against bowtie’s paired results [36] by RSEM (http://deweylab.biostat.wisc.edu/rsem/). Differential expression analyses among the two treatments (CKL-vs.-M2L and CKF-vs.-M2F with three biological replicates per treatment) were conducted using edgeR software (Version 3.24.3) [37]. Genes with a fold change ≥ 2 and a false discovery rate (FDR) < 0.05 in a comparison were defined as significant DEGs. DEGs were then subjected to enrichment analysis of GO functions and KEGG pathways. Heatmaps of DEGs were analyzed using HemI [38].

### 2.6. Quantitative Real-Time PCR (qRT-PCR)

The RNA samples (1 μg) with A260/A230 ratios between 1.8 and 2.2 were used to synthesize the first strand of cDNA with the First Strand cDNA Synthesis Kit (CISTRO, Guangzhou, China) in a 20 μL reaction mixture according to the manufacturer’s protocol. The cDNA samples were diluted 10-fold with distilled water prior to the qRT-PCR analysis. The qRT-PCR analysis was performed using ChamQ Universal SYBR qPCR Master Mix (Vazyme, Nanjing, China) and Roche Light Cycle96 Real-Time PCR system with the following reaction conditions: initial denaturation at 95 °C for 30 s, followed by a two-step program of 95 °C for 10 s and 60 °C for 30 s for 40 cycles, with a melting curve analysis at 95 °C for 15 s, then from 60 °C to 95 °C at a rate of 0.2 °C/s. Candidate genes related to phillyrin were identified from *F. suspensa* transcriptome data, the longest CDS sequence was selected, and the specific primers for the target genes were designed using Oligo 7.0, and are listed in Table S1. The *UKN1* + *SDH* + *G6PD* genes were used as reference gene group and the relative expression levels were determined according the 2^−ΔΔCt^ method [39,40]. The specificity of the primer pair was verified by the presence of a single peak in the melt curve analysis during the qRT-PCR process (Figure S1). All biological replicates were performed in triplicate.

### 2.7. Statistical Analyses

Data were analyzed with one-way ANOVA by using SPSS 17.0, and means were contrasted with Duncan’s multiple scope test at *p* < 0.05. All the experiments were carried out in triplicate.

## 3. Results

### 3.1. Effect of MeJA Induction on the Content of Phillyrin

**Figure 1:**
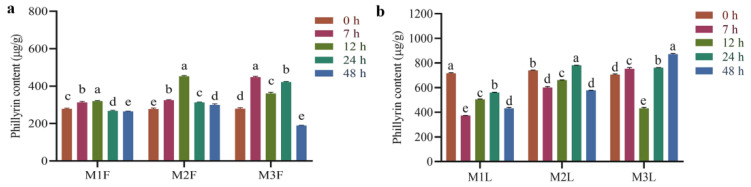
Phillyrin content of *F. suspensa* fruits and leaves treated with different MeJA. (**a**) Phillyrin content of *F. suspensa* fruits treated with different MeJA. (**b**) Phillyrin content of *F. suspensa* leaves treated with different MeJA. M1F: *F. suspensa* fruit treated with 50 µM MeJA; M2F: *F. suspensa* fruit treated with 200 µM MeJA; M3F: *F. suspensa* fruit treated with 400 µM MeJA. M1L: *F. suspensa* leaf treated with 50 µM MeJA; M2L: *F. suspensa* leaf treated with 200 µM MeJA; M3L: *F. suspensa* leaf treated with 400 µM MeJA. Data were analyzed by SPSS, followed by Duncan’s honestly significant difference test at *p* < 0.05. All Statistical analyses of data had three biological repeats. All data are displayed as means ± SD.

The effect of MeJA treatment on the content of phillyrin in the leaves and fruits of *F. suspensa* was shown in Figure 1. After 50 μM MeJA treatment, the phillyrin content in *F. suspensa* fruits was slightly increased compared with the control. Meanwhile, the content of phillyrin was gradually increased and then decreased after 200 μM MeJA treatment, peaking at 12 h with 1.63-fold compared with control. After 400 μM MeJA treatment, the content of phillyrin was obviously increased at 7 h and 24 h with 1.60 and 1.51-fold compared with control, respectively (Figure 1a). After 50 μM MeJA treatment of *F. suspensa* leaves, the content of phillyrin decreased compared to the control. After 200 μM MeJA treatment, the content of phillyrin decreased, then increased, and finally decreased with time. 400 μM MeJA treatment of *F. suspensa* leaves showed a decrease in phillyrin content at 12 h compared with the control (Figure 1b). This indicates that different parts of *F. suspensa* responded differently to the induction of different MeJA concentrations. The exogenous MeJA treatment of *F. suspensa* led to the induction of phillyrin in response to MeJA, but the induction effect varied among time and tissue parts. Due to *F. suspensa* leaves having been used to make health-care tea in folk tradition [8], we also treated them with 200 μM MeJA for 12 h to investigate the different mechanisms of phillyrin synthesis in *F. suspensa* leaves and fruits, under the same conditions of treatment.

### 3.2. Illumina Sequencing and De Novo Assembly

To further understand the molecular mechanism underlying the response of phillyrin to MeJA, an RNA-seq analysis was performed. We established 12 cDNA libraries for transcriptome sequencing. Illumina Hiseq 6000 platform generated 98.21 Gb clean reads with an average Q30 value of 94.55% and an average GC content of 44%, Q20 > 98%, and Q30 > 94% (Table 1). The max length, min length, and N50 length, for the assembled unigenes were 15,856, 201, and 1851 bp, respectively. With regards to the length distribution of the unigenes, the total number of sequences of all unigene lengths of 200–500 bp in the transcriptome data of *F. suspensa* is much higher than the total number of sequences of other respective lengths (Figure S2).

All of the sequencing data were deposited in the National Center for Biotechnology Information (NCBI). The sequence read archive (SRA) accession number is SRP392325. The resulting fastq files were deposited on SRR21066404, SRR21066405, SRR21066406, SRR21066407, SRR21066412, SRR21066413 (leaf) and SRR21066408, SRR21066409, SRR21066410, SRR21066411, SRR21066414, SRR21066415 (fruit).

### 3.3. Functional Annotation of Unigenes

A total of 37,297, 25,361, 15,161, 30,017, 29,688, and 24,539 unigenes had significant hits (E-value ≤ 10^−5^) in the Nr (non-redundant protein) database, Swissprot, Kyoto Encyclopedia of Genes and Genomes (KEGG), the COG database and the Pfam database, respectively (Figure S3). A total of 60,093 CDS unigenes produced by the Illumina Hiseq 2500 platform, and most CDSs, were less than 1800 bp (Figure S4).

**Figure 2:**
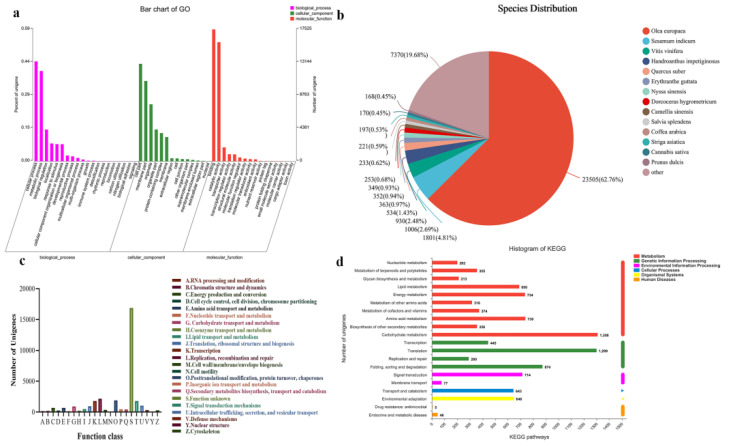
Functional annotation of *F. suspensa* transcriptome. (**a**) Gene Ontology (GO) functional classification of assembled unigenes. (**b**) Homologous species distribution of *F. suspensa* annotated in the NR database. (**c**) Clusters of Orthologous Groups (COG) functional classifification of assembled unigenes. (**d**) Functional classifification and pathway assignment of assembled unigenes by Kyoto Encyclopedia of Genes and Genomes (KEGG).

For GO annotation, 79,094 unigenes were allocated into three categories (“biological process”, “cellular component”, and “molecular function”). Among the biological processes, the unigenes were mainly involved in metabolic processes (8180, GO: 0008152), cellular processes (9058, GO: 0009987), and biological regulation (2432, GO: 0065007). Among the cellular components category, the unigenes were primarily associated with cellular fraction (7846, GO: 0044464), membrane fraction (7269, GO: 0044425), and organelles (4424, GO: 0043226). Among the molecular function categories, unigenes with catalytic activity (10,376, GO: 0003824), binding (12,606, GO: 0005488), and transporter protein activity (1323, GO: 0005215), accounted for a larger proportion (Figure 2a).

Unigene sequences were searched against the NR database for annotation and revealed 23,505 unigenes (62.76%) matched to *Olea europaea* and 1801 unigenes (4.81%) matched to *Sesamum indicum* (Figure 2b).

To further evaluate the completeness of the transcriptome library and the validity of the annotation, the COG classification of unigenes was performed. Among the 23 COG categories, “L: Replication, recombination and repair” was the largest group, followed by “O: Posttranslational modification, protein turnover, chaperones and ion, protein turnover, chaperones” and “T: Signal transduction mechanisms”. The smallest group was “Y: Nuclear structure” with only two unigenes annotated to this category (Figure 2c).

The KEGG database was used to identify the biochemical pathways assigned to unigene sequences. In our results, a total of 10,325 unigenes were annotated to six metabolic pathways: metabolism, genetic information processing, environmental information processing, cellular processes, organismal systems, and human diseases. Metabolism had the largest number of pathways and human diseases had the smallest number of pathways. There were 356 unigenes matching to the biosynthesis of other secondary metabolism, and 224 unigenes of phenylpropanoid biosynthesis pathway (Figure 2d).

### 3.4. Analysis of Differentially Expressed Unigenes (DEGs)

**Figure 3:**
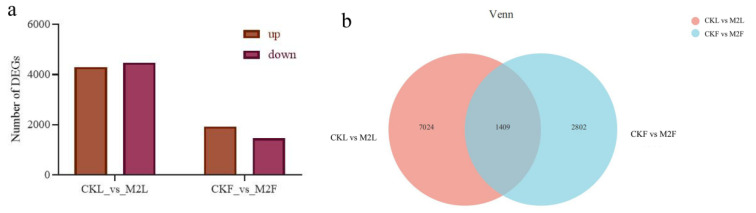
Statistics of DEGs in *F. suspensa* transcriptomes. (**a**) DEG numbers in comparisons of the CKF-vs.-M2F and CKL-vs.-M2L. (**b**) Venn diagram showing significantly DEGs at different tissue. CKL: *F. suspensa* leaf treated with control solution for 0 h; M2L: *F. suspensa* leaf treated with 200 µM MeJA; CKF: *F. suspensa* fruit treated with control solution for 0 h; M2F: *F. suspensa* fruit treated with 200 µM MeJA.

To obtain a comprehensive view of the gene expression profile associated with the response of phillyrin to MeJA, we used edgeR to identify the DEGs. A total of 4211 (2348 up-regulated and 1863 down-regulated) DEGs and 8433 (4134 up-regulated and 4299 down-regulated) DEGs were identified in fruits and leaves of *F. suspensa* treated 12 h with MeJA, respectively (Figure 3); this finding indicated that there were more upregulated than downregulated unigenes in *F. suspensa* fruits and more downregulated than upregulated unigenes in *F. suspensa* leaves under MeJA treatment. Furthermore, we used principal component analysis (PCA) to display the overall relationships between the transcriptome from the different samples (Figure S5), indicating the leaves and fruits of *F. suspensa* before and after MeJA treatment showed mutual aggregation within the group. Meanwhile, two different transcriptional variation tendencies were observed before and after the MeJA treatment of *F. suspensa* fruits, and *F. suspensa* leaves also showed the same trend.

**Figure 4:**
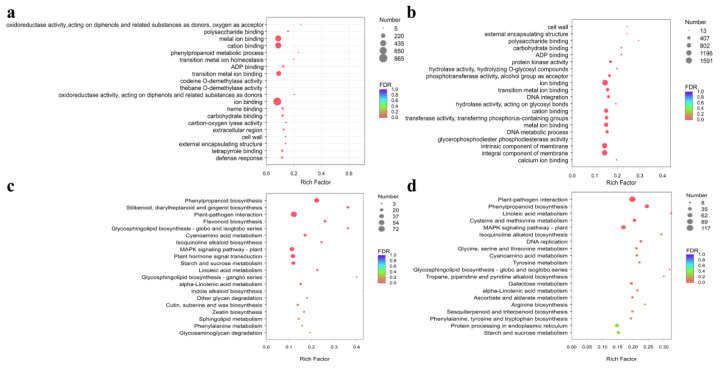
Enrichment analysis of differentially expressed genes in the GO and KEGG pathways. (**a**) GO pathway enrichment analysis in CKF-vs.-M2F. (**b**) GO pathway enrichment analysis in CKL-vs.-M2L. (**c**) KEGG pathway enrichment analysis in CKF-vs.-M2F. (**d**) KEGG pathway enrichment analysis in CKL-vs.-M2L. CKL: *F. suspensa* leaf treated with control solution for 0 h; M2L: *F. suspensa* leaf treated with 200 µM MeJA; CKF: *F. suspensa* fruit treated with control solution for 0 h; M2F: *F. suspensa* fruit treated with 200 µM MeJA.

Functional enrichment analysis revealed that 3754 and 1822 DEGs were associated with GO terms in *F. suspensa* leaves and fruits, respectively (Figure 4). DEGs were mainly enriched in oxidoreductase activity (GO: 0016682), and phenylpropanoid metabolic process (GO: 0009698) (Figure 4a,b). The KEGG pathways analysis indicated DEGs were mainly involved in the metabolism of phenylpropanoid biosynthesis, plant-pathogen interaction, MAPK signaling pathway-plant, phenylalanine metabolism, and plant hormone signal transduction (Figure 4c,d).

**Figure 5:**
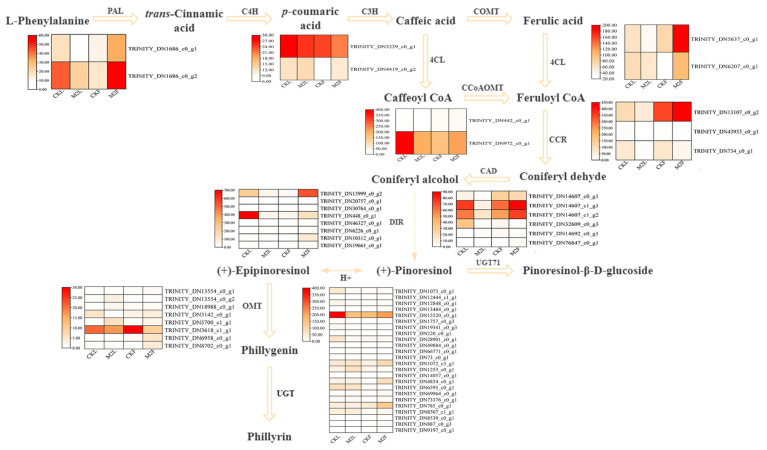
Putative pathway for phillyrin synthesis in *F. suspensa*. PAL, phenylalanine ammonia-lyase; C3H, Coumarate 3-hydroxylase; 4CL, 4-coumarate CoA ligase; CCoAOMT, caffeoyl-CoA O-methyltransferase; CCR, cinnamoyl-CoA reductase; CAD, cinnamyl alcohol dehydrogenase; DIR, dirgent protein; OMT, O-methyltransferase; UGT, UDP-glycosyltransferase. CKL: *F. suspensa* leaf treated with control solution for 0 h; M2L: *F. suspensa* leaf treated with 200 µM MeJA; CKF: *F. suspensa* fruit treated with control solution for 0 h; M2F: *F. suspensa* fruit treated with 200 µM MeJA.

Previous study showed that phillyrin as a kind of lignan was tightly related to phenylpropanoid biosynthesis [41]. In the phillyrin biosynthesis pathway, three *FsCCR*, four *FsCAD*, six *FsDIR*, five *FsOMT*, one *FsPAL*, and thirteen *FsUGT* genes were significant different after MeJA treatment in *F. suspensa* leaves, respectively (Figure 3 and Figure 5). Meanwhile, two *Fs4CL*, two *FsCAD*, one *FsCCR*, four *FsDIR*, three *FsOMT*, two *FsPAL*, and twelve *FsUGT* were significant different in *F. suspensa* fruits. Furthermore, one *FsUGT*, three *FsOMT*, two *FsDIR*, one *FsPAL*, and one *FsCCR* were significant different both in *F. suspensa* leaves and fruits (Figure 3 and Figure 5). These results suggest that MeJA may induce the accumulation of phillyrin by regulating the expression of genes associated with phillyrin biosynthesis in *F. suspensa*.

### 3.5. Analysis of DEGs Related to Phillyrin Biosynthetic Pathway

**Figure 6:**
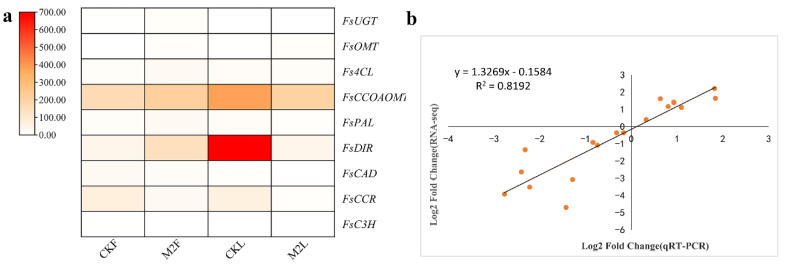
Expression patterns of genes related to phillyrin biosynthesis. (**a**). Transcriptional profiles of DEGs related to phillyrin biosynthetic pathway. (**b**). Scatter plot and linear regression based on qRT-PCR and RNA-seq. The correlation coefficient was calculated using the Spearman correlation method. *FsPAL*, *phenylalanine ammonia-lyase gene*; *FsC3H*, *Coumarate 3-hydroxylase gene*; *Fs4CL, 4-coumarate CoA ligase gene*; *FsCCoAOMT*, *caffeoyl-CoA O-methyltransferase gene*; *FsCCR*, *cinnamoyl-CoA reductase gene*; *FsCAD*, *cinnamyl alcohol dehydrogenase gene*; *FsDIR*, *dirgent protein gene*; *FsOMT*, *O-methyltransferase gene*; *FsUGT*, *UDP-glycosyltransferase gene.* CKL: *F. suspensa* leaf treated with control solution for 0 h; M2L: *F. suspensa* leaf treated with 200 µM MeJA; CKF: *F. suspensa* fruit treated with control solution for 0 h; M2F: *F. suspensa* fruit treated with 200 µM MeJA.

To verify the expression level of genes related to phillyrin biosynthesis exhibited by the identified DEGs in response to MeJA, the expression levels of nine genes (*FsUGT*, *Fs4CL*, *FsCCoAOMT*, *FsOMT*, *FsPAL, FsDIR, FsCAD*, *FsCCR*, and *FsC3H*) were detected by qRT-PCR. The expression profiles were generally consistent with the RNA-seq results (Figure 6a). Correlation analysis based on the qRT-PCR and transcriptome data also showed a significant correlation with a Spearman correlation coefficient of 0.819, which reflects the accuracy of transcriptome data (Figure 6b). To analyze the dynamic expression profiles of genes putatively related to phillyrin synthesis under MeJA treatment, genes expression level (*FsUGT*, *Fs4CL*, *FsCCoAOMT*, *FsOMT*, *FsPAL*, *FsDIR*, *FsCAD*, *FsCCR*, and *FsC3H*) in *F. suspensa* fruits and leaves after MeJA treatment in different time (0 h, 7 h, 12 h, and 24 h) were studied. The results revealed that MeJA activated or suppressed the expression of genes to variant degrees at different time points and in different tissues.

**Figure 7:**
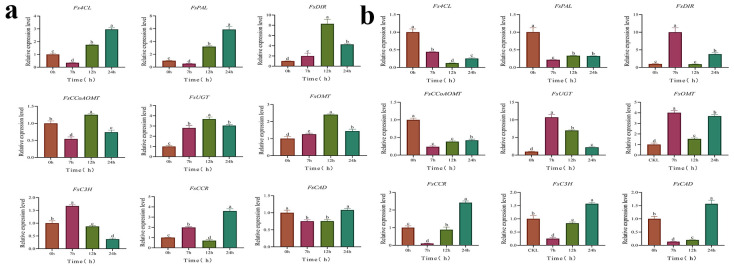
Analysis of the expression profiles of nine genes related to phillyrin synthesis after 200 µM MeJA treatment for 0, 7, 12, and 24 h. (**a**). Relative expression levels of phillyrin biosynthesis genes in *F. suspensa* fruits are detected by qRT-PCR (0 h as 1). (**b**). Relative expression levels of phillyrin biosynthesis genes in *F. suspensa* leaves are detected by qRT-PCR (0 h as 1). The *UKN1* + *SDH* + *G6PD* genes are used as the reference gene group to standardize the RNA samples for each reaction. All Statistical analyses of data had three biological repeats. Data are analyzed by SPSS, followed by Duncan’s honestly significant difference test at *p* < 0.05. All Statistical analyses of data had three biological repeats. *FsPAL*, *phenylalanine ammonia-lyase gene*; *FsC3H*, *Coumarate 3-hydroxylase gene*; *Fs4CL, 4-coumarate CoA ligase gene*; *FsCCoAOMT*, *caffeoyl-CoA O-methyltransferase gene*; *FsCCR*, *cinnamoyl-CoA reductase gene*; *FsCAD*, *cinnamyl alcohol dehydrogenase gene*; *FsDIR*, *dirgent protein gene*; *FsOMT*, *O-methyltransferase gene*; *FsUGT*, *UDP-glycosyltransferase gene*.

The expression of *FsDIR*, *FsUGT*, and *FsOMT* genes were up-regulated at all time points in 200 μM MeJA-treated *F. suspensa* fruit. In the presence of MeJA, the expression levels of *Fs4CL*, *FsPAL*, *FsCCR,* and *FsCAD* genes, were inhibited at the earlier time points and activated at the later time points. The expression level of *FsCCoAOMT* gene reached a maximum at 12 h, and then its expression decreased compared with the 0 h control. The expression levels of *FsC3H* gene reached a maximum at 7 h and were repressed thereafter compared with the 0 h control (Figure 7a). In *F. suspensa* leaves, *Fs4CL*, *FsPAL*, and *FsCCoAOMT* genes expression levels were down-regulated at all time points, and *FsOMT* and *FsUGT* genes expression were up-regulated within 24 h of MeJA treatment compared with the 0 h control. *FsCCR*, *FsC3H*, and *FsCAD* genes expression were repressed at early time points, afterwards, expression gradually increased (Figure 7b).

## 4. Discussion

The content of phillyrin is an important index for evaluating the quality of *F. suspensa*. Phillyrin are effective in clearing heat and detoxifying, reducing swelling, and dispersing nodules [42]. It is important to further understand the phillyrin biosynthesis pathway and provide robust candidate genes for further functional investigations aimed at the improvement of phillyrin content and quality.

MeJA is a cyclopentanone derivative-like signaling substance commonly found in the plant kingdom that regulates plant growth and development, triggers cells to initiate protective mechanisms, and stimulates the expression of key enzymes of metabolic pathways [43]. Plant defense systems are initiated upon injury or by signals from exogenous plant hormones, such as MeJA. Moreover, lignans, at least in part, are believed to be involved in host defense systems [44,45,46]. In combination, the hormone is expected to enhance lignan biosynthesis [45,47]. We also found that MeJA could induce the accumulation of phillyrin in *F. suspensa* fruit (Figure 1a), but a certain concentration of MeJA could inhibit the synthesis of phillyrin in *F. suspensa* leaves (Figure 1b). The content of phillyrin in leaves without MeJA treatment was higher than that in fruits without MeJA treatment, which was consistent with the results of previous studies [48]. The efficacy of *F. suspensa* leaves recorded in traditional Chinese medicine classics is very similar to that of *F. suspensa* fruits, and the chemical composition is also similar [49]. According to the theory of traditional Chinese medicine, the effects of different parts of *F. suspensa* should be completely different, therefore, we performed RNA-seq detection on *F. suspensa* fruits and leaves to compare the molecular mechanism of phillyrin biosynthesis in *F. suspensa* fruits and leaves, laying a foundation for the comprehensive development and utilization of *F. suspensa* resources.

GO and KEGG results showed DEGs were mainly enriched in phenylpropanoid metabolic process in *F. suspensa* after MeJA treatment (Figure 4), the phenylpropanoid pathway, a common pathway of phillyrin biosynthesis pathway, can produce coniferyl alcohol, which are precursors of phillyrin [50], including *Fs4CL*, *FsCCR*, *FsCAD*, *FsC3H*, *FsCCoAOMT*, and *FsPAL* genes, which were significantly different in *F. suspensa* after MeJA treatment compared with control (Figure 3 and Figure 5). Research shows that during phillyrin biosynthesis, two coniferyl alcohol molecules are coupled to produce compounds of different configurations, and then converted to (+)-epipinoresinol. Furthermore, the (+)-epipinoresinol is converted to phillygenin, eventually forming phillyrin [23], including the *FsDIR, FsOMT,* and *FsUGT* gene, which were noticeably different in *F. suspensa* after MeJA treatment compared with control. When *F. suspensa* fruits were treated with different concentrations of MeJA, the content of phillyrin in the fruits increased, and most of the genes in its synthesis pathway showed an increasing trend; the expression of *Fs4CL*, *FsCCR*, *FsCAD*, *FsC3H*, *FsCCoAOMT*, and *FsPAL* in MeJA-treated *F. suspensa* leaves was suppressed for a certain period of time, while the expression of *FsOMT* and *FsUGT* increased, and phillyrin was also decreased (Figure 1 and Figure 7). Different parts of *F. suspensa* have different responses to MeJA induction, which has been reported in other plants [51]. This may indicate that genes, during the synthesis of the lignan precursor substance coniferyl alcohol, also have an important influence on the synthesis of the end product phillyrin; and that there are complex regulatory mechanisms involved in the transcription of genes to translation into proteins, from the processing and modification of proteins into active enzymes to secondary metabolites and accumulation.

The fruit of *F. suspensa* is the main part of the medicine; our study showed that MeJA treatment could effectively increase the content of phillyrin in *F. suspensa* fruits (Figure 1). The expression of *FsOMT* and *FsUGT* was up-regulated in both the fruits and leaves of *F. suspensa* after MeJA treatment. Previous study showed that *Lycoris aurea LaOMT1* transcripts were significantly increased after MeJA treatment [52]; *Bupleurum chinense DC.* glycosyltransferase genes *BcUGT1*, *BcUGT3,* and *BcUGT6* could be induced by MeJA with different degrees of up-regulation of expression [53]. The expression of *FsCAD*, *FsCCR,* and *FsC3H* did not correspond to the phillyrin content in *F. suspensa* fruit, and the expression of *FsOMT* and *FsUGT* did not correspond to the phillyrin content in *F. suspensa* leaves. The result was not a surprise because the formation of end products is collaborative with all genes in the synthesis pathway. The present study cannot adequately account for the role of these individual genes in phillyrin biosynthesis. Therefore, it is crucial to establish a transgenic system for *F. suspensa* in order to further confirm the function of genes in the phillyrin synthesis pathway. Recently, our team has been making a concerted effort to establish a system for transgenic *F. suspensa* with an emphasis on hairy roots and callus. In this transgenic system, a deeper understanding of the molecular mechanisms of phillyrin will be gained, and the biotechnological improvement of *F. suspensa*.

## 5. Conclusions

In this study, using de novo sequencing, a *F. suspensa* dataset containing 87,564 unigenes was constructed to molecular mechanism underlying the response of phillyrin to MeJA in *F. suspensa* leaves and fruits. A total of 4211 and 8433 DEGs were identified in the fruits and leaves of *F. suspensa* treated with MeJA for 12 h, respectively. GO and KEGG analyses indicated that DEGs were mainly enriched in phenylpropanoid metabolic process in *F. suspensa*, which was tightly associated with phillyrin biosynthesis. The qRT-PCR results showed that *FsUGT*, *Fs4CL*, *FsCCoAOMT*, *FsOMT*, *FsPAL*, and *FsDIR* were significantly up-regulated in *F. suspensa* fruits after MeJA treatment; the corresponding *F. suspensa* fruit also increased the content of phillyrin. While *FsCAD Fs4CL*, *FsCCoAOMT*, *FsCCR*, *FsPAL*, *FsC3H,* and *FsDIR* were significantly down-regulated in *F. suspensa* leaves, the content of phillyrin in *F. suspensa* leaves with the same treatment was reduced compared with that of the control. These qRT-PCR data were consistent with RNA-seq data. Taken together, the analysis of transcriptome results of *F. suspensa* leaves and fruits lay a foundation for the comprehensive development and utilization of *F. suspensa* resources. These results provide a robust theoretical basis for accelerating the study of the regulatory mechanism of MeJA-induced phillyrin biosynthesis in *F. suspensa*.

## Figures and Tables

**Table 1:** Summary of sequencing and assembly for *F. suspensa*.

Type	Unigene
Total number	87,564
Total base	87,582,604
Largest length (bp)	15,856
Smallest length (bp)	201
Average length (bp)	1000.21
GC percent (%)	44
N50 average length (bp)	1851
Q_30_ (%)	94.55
Q_20_ (%)	98

## Data Availability

The datasets presented in this study can be found in online repositories. The sequencing data can be found in the National Center for Biotechnology Information (NCBI) Sequence Read Archive under accession number PRJNA869193.

## Supplementary Materials

The following supporting information can be downloaded at: https://www.mdpi.com/article/10.3390/metabo12111143/s1, Table S1: qRT-PCR primer information. Figure S1: Melt curve of representative qRT-PCR amplicons. Figure S2: The sequence length distribution of assembled unigenes. Figure S3: Gene function annotations in six databases (GO, KEGG, COG, NR, Swiss-Prot, Pfam) of *F. suspensa*. Figure S4: Length distribution of CDS in unigenes. Figure S5: Principal component analysis of expressed gene. CKL: *F. suspensa* leaf treated with control solution for 0 h; M2L: *F. suspensa* leaf treated with 200 µM MeJA; CKF: *F. suspensa* fruit treated with control solution for 0 h; M2F: *F. suspensa* fruit treated with 200 µM MeJA.
