# Supplementary material for: Comparative Transcriptome Analysis of MeJA Responsive Enzymes Involved in Phillyrin Biosynthesis of Forsythia suspensa
Source: Metabolites. 2022 Nov 20;12(11):1143. doi: 10.3390/metabo12111143 (PMC9694870; doi:10.3390/metabo12111143)
Supplement: Supplementary file 1 [file metabolites-12-01143-s001.zip › metabolites-2015873-supplementary/Table S1.pdf]

Supplementary Table S1. qRT-PCR primer information

Gene Name	Unigene name	amplicon size（bp）	Primer name	Primer sequences（5’-3’）
FsUGT	TRINITY_DN 13484_c0_g1	239	qUGT-F	AGCTTGTGGTCTGAATCGGA
			qUGT-R	AGCCCTCGATCCTTCATACG
FsOMT	TRINITY_DN 12053_c0_g1	153	qOMT-F	ATGCGAAAATTTGCAGGGAAG
			qOMT-R	CCAGCAGCAGGAAAAAAGAGA
FsCCoAOMT	TRINITY_DN 972_c0_g1	180	qCCoAOMT-F	CAAATGATTGAAGATGGGAAGA
			qCCoAOMT-R	CAATGGTGCATCAGGTGG
FsDIR	TRINITY_DN 448_c0_g1	196	qDIR-F	TCAGGCGATTGACAAACAGC
			qDIR-R	ATCCATAACAAAGACCGCGC
FsC3H	TRINITY_DN 4745_c0_g2	264	qC3H-F	GGAGCCCATCAAGGAGCAAT
			qC3H-R	TGGCATCTTCCGAGTGTGTG
Fs4CL	TRINITY_DN 9140_c0_g2	156	q4CL-F	GAAGTCGGCTGATGTGGTTC
			q4CL-R	ACGCGGTACCTCTGTATCAG
FsCAD	TRINITY_DN 14607_c0_g1	150	qCAD-F	CCACCCTCTTGAGCCTTAC
			qCAD-R	TCCTTGATGCTCCCGATAA
FsCCR	TRINITY_DN 734_c0_g1	184	qCCR-F	GCAGCAGTATGGAGACAAA
			qCCR-R	GACCAGGATTGATGGAAAC
FsPAL	TRINITY_DN 1686_c0_g1	117	qPAL-F	GGCTGCTCAGAAACTACACG
			qPAL-R	CACTTCAATTTGTGGGCCGA
UKN1 (Shen, et al., 2020)		92	qUKN1-F	CAGACCAGCTTTGAGGAGTATC
			qUKN1-R	GGCCAGAAACCAGTAGTCAATA
SDH (Shen, et al., 2020)		100	qSDH-F	GAAATCGTCCCAACCTCTTACC
			qSDH-R	AGCTGTTCAGGACAAGGTTATG
G6PD (Shen, et al., 2020)		119	qG6PD-F	TGCTCGGGCTTGATAGAAAC
			qG6PD-R	GAAGGACGCGGTGGATATTT
