# Supplementary figures and images for: Comparative Transcriptome Analysis of MeJA Responsive Enzymes Involved in Phillyrin Biosynthesis of Forsythia suspensa
Source: Metabolites. 2022 Nov 20;12(11):1143. doi: 10.3390/metabo12111143 (PMC9694870; doi:10.3390/metabo12111143)

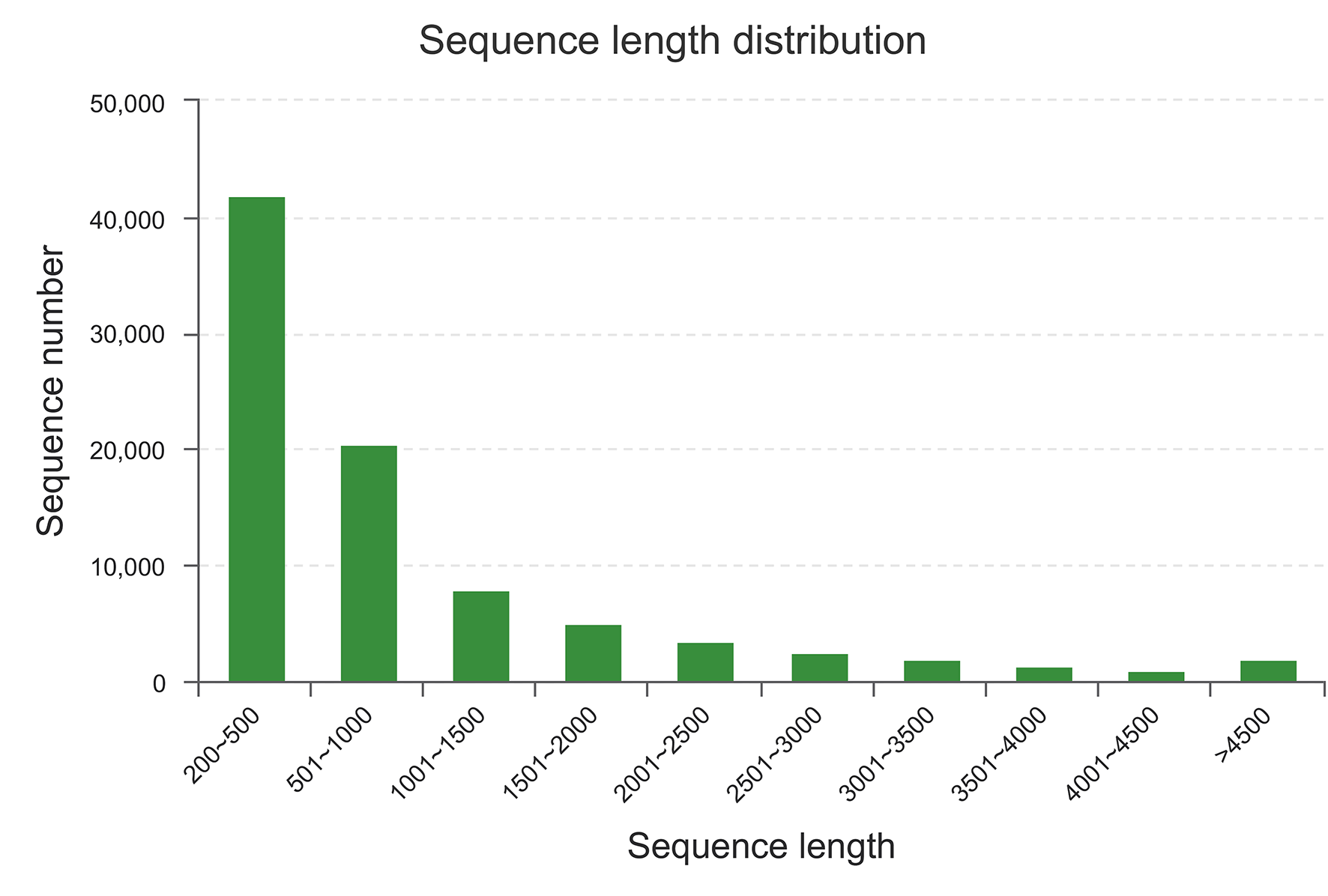

Supplement: Supplementary file 1 [file metabolites-12-01143-s001.zip › metabolites-2015873-supplementary/Figure S2.tif]

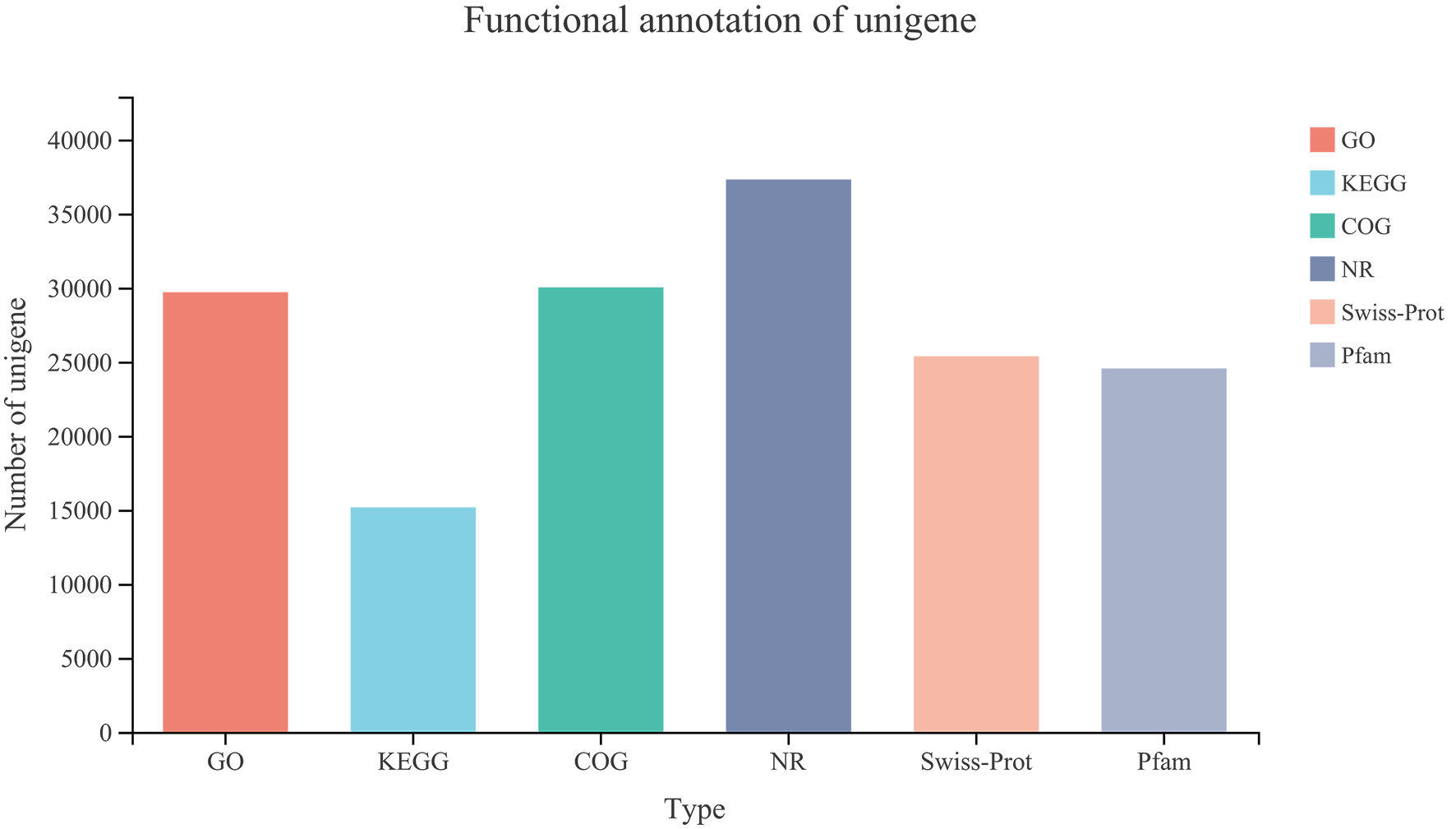

Supplement: Supplementary file 1 [file metabolites-12-01143-s001.zip › metabolites-2015873-supplementary/Figure S3.tif]

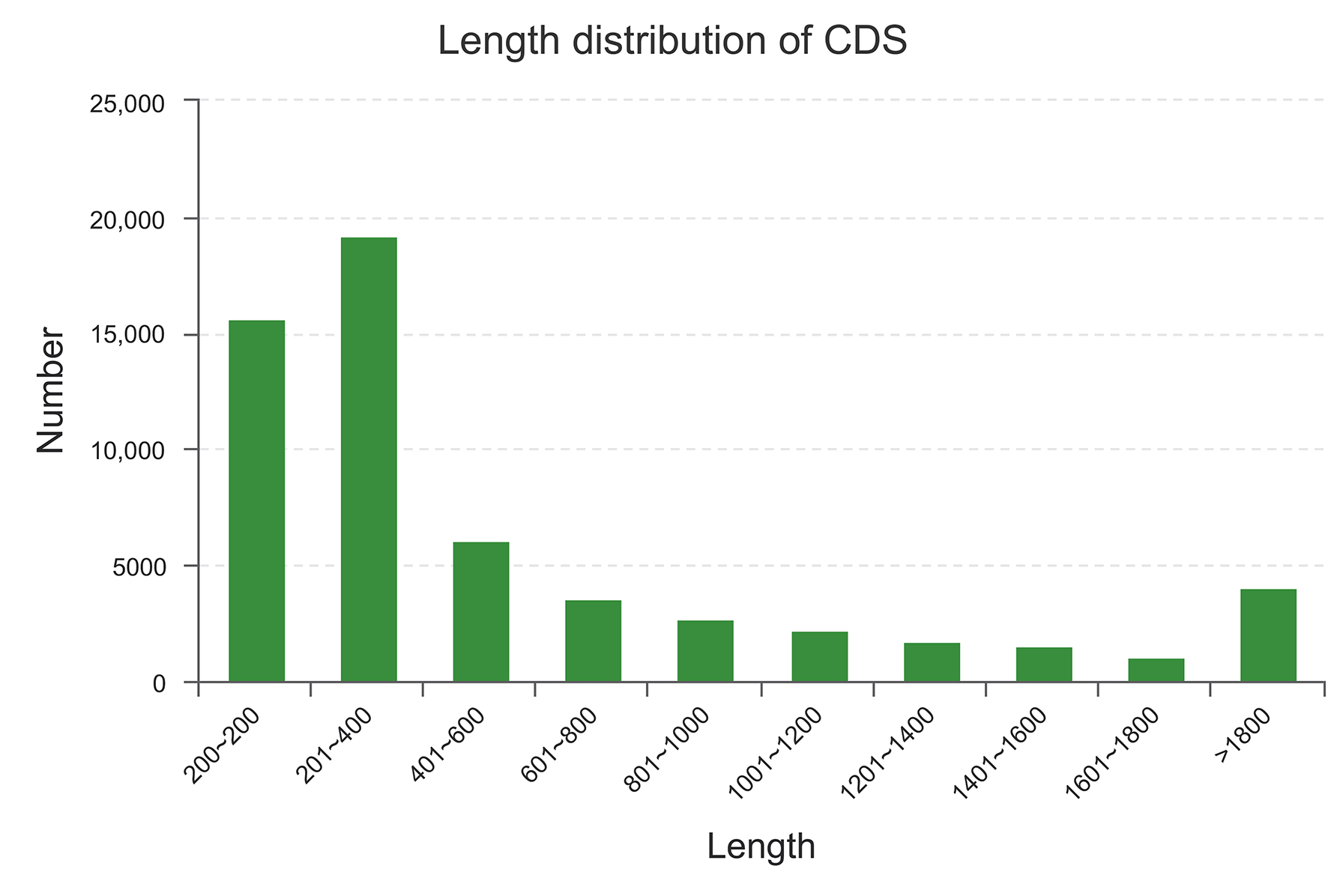

Supplement: Supplementary file 1 [file metabolites-12-01143-s001.zip › metabolites-2015873-supplementary/Figure S4.tif]

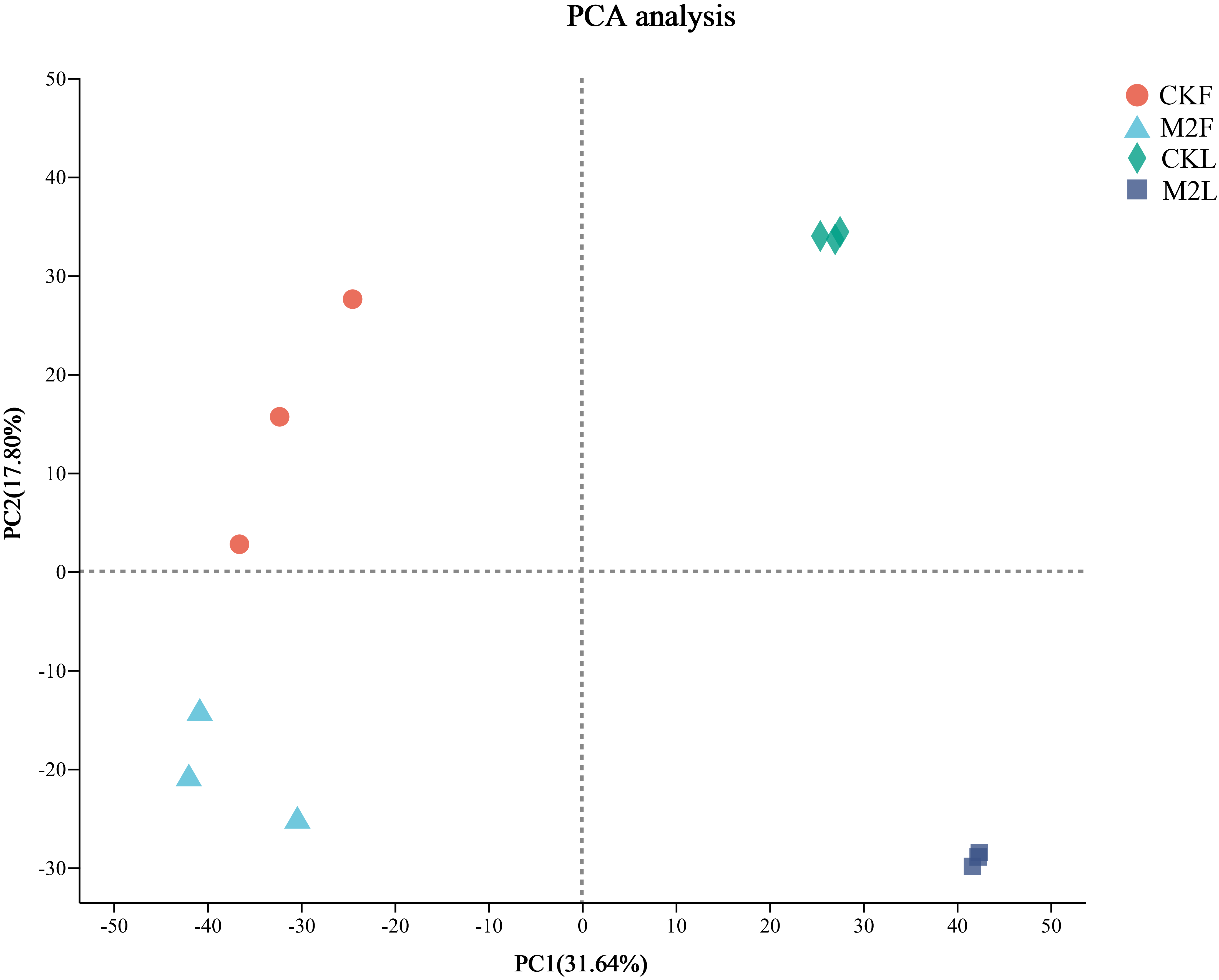

Supplement: Supplementary file 1 [file metabolites-12-01143-s001.zip › metabolites-2015873-supplementary/Figure S5.tif]
